# State-of-Health Prediction Using Transfer Learning and a Multi-Feature Fusion Model

**DOI:** 10.3390/s22218530

**Published:** 2022-11-05

**Authors:** Pengyu Fu, Liang Chu, Zhuoran Hou, Zhiqi Guo, Yang Lin, Jincheng Hu

**Affiliations:** 1 College of Automotive Engineering, Jilin University, Changchun 130022, China; 2 Department of Aeronautical and Automotive Engineering, Loughborough University, Loughborough LE11 3TU, UK

**Keywords:** state of health (SOH), convolutional neural network (CNN), long short-term memory (LSTM), transfer learning (TL), multi-feature fusion (MFF)

## Abstract

Existing data-driven technology for prediction of state of health (SOH) has insufficient feature extraction capability and limited application scope. To deal with this challenge, this paper proposes a battery SOH prediction model based on multi-feature fusion. The model is based on a convolutional neural network (CNN) and a long short-term memory network (LSTM). The CNN can learn the cycle features in the battery data, the LSTM can learn the aging features of the battery over time, and regression prediction can be made through the full-connection layer (FC). In addition, for the aging differences caused by different battery operating conditions, this paper introduces transfer learning (TL) to improve the prediction effect. Across cycle data of the same battery under 12 different charging conditions, the fusion model in this paper shows higher prediction accuracy than with either LSTM and CNN in isolation, reducing RMSPE by 0.21% and 0.19%, respectively.

## 1. Introduction

In recent years, to alleviate energy consumption, environmental pollution, global warming, and other issues, multinational governments have vigorously developed electric vehicles (EVs) to build a clean, economic, efficient, and environmentally friendly society [1,2,3]. With the continuous improvement of technology, EVs have gained stronger performances and service lives and have gained more and more recognition from consumers. However, the high power consumption of medium and large EVs is not conducive to the realization of energy conservation and emission reduction goals. In addition to the design, manufacturing, and process of battery, a battery management system (BMS) can be used to control the charging and discharging process of power batteries; this is an easy way to reduce power consumption [4]. With the use of a power battery, its capacity and power will inevitably decline. Therefore, accurate prediction of the state of health (SOH) is an important function of the BMS that can provide a reference for battery control strategies [5].

However, SOH is the internal status of the battery, which is difficult to directly measure and obtain [6,7,8]. Previously, the experimental method was the mainstream detection method, but it was difficult to apply it to online detection. At the same time, this requires a lot of manpower and testing costs, and there are short circuit and other risk factors, leading to accidents.

In addition to detection methods, SOH prediction methods can be divided into two subcategories: model-driven methods and data-driven methods. Model-driven methods are highly explanatory and accurate. However, the process of model establishment is complex, and it is difficult to identify the parameters of the model. Following the improvement of data collection technology, more attention has been given to data-driven methods without battery models.

Data-driven methods do not need to study the internal aging mechanism of the battery; they only need to determine the nonlinear mapping relationship between the aging features of the battery and the battery SOH. These are mainly divided into machine learning methods that need to manually filter features and deep learning methods that automatically filter features on the network.

Although these data-driven methods can produce satisfactory accuracy of SOH prediction, they all have obvious defects. The machine learning methods are weak in extracting battery cycle features, so it is necessary to manually extract battery cycle features. Moreover, the extracted battery cycle features are not necessarily suitable for the prediction of battery SOH. Many articles choose appropriate evaluation methods to evaluate the correlation between battery cycle features and SOH. The deep learning methods only obtain the features in the battery cycle and ignore the impact of battery history information, and the battery data will be greatly affected when there is noise interference. To alleviate the above problems, this paper proposes a battery SOH prediction model based on multi-feature fusion, which extracts battery cycle features and battery aging features at the same time to achieve an accurate prediction of battery SOH. The major contributions of this work are as follows:

1. A multi-feature fusion model is proposed for SOH prediction, which can simultaneously obtain multiple features related to battery aging, and achieve more accurate prediction;
2. A convolutional neural network (CNN) is used to extract battery cycle features, and battery cycle features no longer rely on manual extraction. The automatic feature extraction process enables a method for training the model through large-scale battery data, reducing the risk of poor applicability of manually extracted features;
3. LSTM is used to extract battery aging features, and the historical cycle features of the battery are introduced. When the battery cycle data are disturbed by noise, the robustness of the model is improved due to the constraints of historical cycle features;
4. Transfer learning is used to improve the prediction accuracy of the target task battery and reduce the training cost of the target task by transferring the features of the source task battery.

The rest of this paper is divided into four parts. Section 2 provides an overview of current SOH prediction approaches. Section 3 introduces the battery test data used in this paper. Section 4 introduces the principle of the method used in this paper and the specific structure of the model. Section 5 presents and explains the validation results. Section 6 summarizes some conclusions and future research directions.

## 2. Related Works

The methods of detecting SOH can be divided into destructive detection methods and non-destructive detection methods according to the experiments. The destructive detection methods require a disassembly experiment of the lithium-ion battery, and the SOH of the lithium-ion battery can be obtained by analyzing the properties of battery modules such as the positive pole, negative pole, diaphragm, electrolyte, etc. [9]. Destructive detection methods can analyze the aging state of each battery component and acquire a strong explanation of the aging mechanism. However, after detection, the damage caused by the disassembly experiment cannot be reversed, which limits the use of the destructive detection method. Nondestructive detection methods do not need to damage the battery structure. The external state closely related to SOH is obtained through actual load charging and discharging experiments [10]. The SOH estimation accuracy of nondestructive detection methods is directly affected by the sampling accuracy of the external state data, so the requirements of testing equipment and testing environment are strict, requiring a lot of labor and testing costs.

Model-driven methods can also be divided into empirical model-based methods [11,12], electrochemical model (EM)-based methods [13,14] and equivalent circuit model (ECM)-based methods [15,16]. Empirical model-based methods do not analyze the mechanism of the actual battery decay process, but analyze mathematical statistics through the data related to the decay and conclude the mathematical relationship between battery parameters and SOH according to the principle of minimum error. Therefore, they are usually used to model the complete cycle of the battery and fit the declining trend of SOH. Han et al. [17] deduced the battery aging model related to battery temperature based on Arrhenius law. The calculation of empirical models is very simple, but the empirical models lack internal mechanisms, and it is difficult to identify reasonable model parameters at the initial stage of prediction. The EM-based methods focus on the internal working principle of the battery and use partial differential equations to describe the charging and discharging behavior of the battery to study the aging mechanism of the battery. The most common EMs are the pseudo-two-dimensional model (P2D model) [18] based on the porous electrode theory and its reduced order models, the single-particle model (SPM) [14], and the lumped-particle model (LPM) [19]. EMs have strong applicability, high accuracy, and strong interpretations. However, EMs usually involve solving large-scale partial differential equations with high complexity, which also makes it difficult to apply EMs to online estimation. The ECM-based methods use common basic circuit components to simulate the working state inside the battery, and the most commonly used are the second-order RC model [20] and fraction-order model [21]. ECMs that fit the mapping of the battery resistance to SOH are called semi-empirical models, which have the advantages of simple structure and small calculation. However, the applicability of ECMs is poor, and it is difficult to identify model parameters.

With the advent of the information age, big data technology has brought opportunities and challenges to the prediction of SOH. Data-driven methods do not need to study the internal aging mechanism of the battery and completely depend on the battery data, which is the current research hotspot. However, obtaining effective data features from large-scale data is the key to predicting SOH. In addition, in the actual operation of the vehicle, the working environment, and the experimental environment of the power battery are very different, which brings great difficulties to data-driven methods.

Machine learning is an artificial intelligence algorithm that can predict SOH by learning the complex dependence of battery features extracted from battery data on SOH [22]. Guo et al. [23] extracted eight effective health features (HFs) from the battery charging curve, obtained indirect health features through principal component analysis (PCA), and established a prediction model of SOH using relevance vector machine (RVM). Li et al. [24] extracted four HFs highly related to SOH decline from the battery charging and discharging data as the input of support vector regression (SVR) and optimized the parameters of the SVR model based on the improved ant lion optimization (ALO) algorithm. Lin et al. [25] selected three HFs from the battery life cycle test data, including the constant current charging time, the instantaneous voltage drop at the start of discharging, and the open-circuit voltage, as the inputs for a capacity estimation probability neural network (PNN) used to predict battery SOH. The above methods use the basic machine learning model. The model structure is relatively simple and can obtain satisfactory accuracy in SOH prediction, but there are some unavoidable limitations. First of all, the applicability of the model is poor because good data and features are prerequisites, and algorithms play a greater role [26]. The models do not have the capability of feature extraction, so feature engineering is required to manually extract features, usually including data preprocessing, feature selection, dimension reduction and so on. Secondly, the factors affecting SOH are diverse, and the manually extracted features may not apply to all battery working conditions. As such, the performance of the same method on different datasets may vary greatly.

Deep learning methods are based on the development of the artificial neural network. The neurons in the artificial neural network can be divided into three different types of layers according to their functions: input layer, hidden layer, and output layer. The neurons contained in each layer can convert the data input to this layer into features and output them to the next layer for further processing. Deep learning methods have more than one hidden layer. Therefore, the underlying features can be combined through multiple hidden layers to form more abstract high-level features. Shen et al. [27] first tried to introduce deep learning into the task of SOH prediction. The voltage, current, and capacity are discretized and input into deep convolutional neural network (DCNN) in the form of a matrix. Compared with the basic machine learning method, it has higher accuracy and robustness. Fan et al. [28] used the fused gate current unit evolutionary neural network (GRU-CNN) model to obtain more features. This model uses a CNN to extract the battery features of the voltage, current and temperature matrix and uses GRU to obtain the time features of these battery data in the order of voltage increase. Finally, a combination of the features of the two networks is used to predict SOH. These methods obtain the features of the charge and discharge cycle of the battery from the data matrix, which can be called methods based on battery cycle features.

The recurrent neural network (RNN) is a kind of deep learning model with short-term memory. The neurons of RNN can not only accept the information from the upper layer of neurons but also can accept their information at the last moment. Therefore, RNN is suitable for mining temporal correlation in sequential data. Eddahech et al. [29] used RNN to accurately predict two SOH-related parameters of the battery, namely capacity and equivalent series resistance. Seven battery HFs, such as temperature, current, and SOC change, are used as input of RNN. The long short-term memory (LSTM) network is a variant of the RNN. Through three new gating mechanisms and a transmission state, LSTM improves the long-term dependence of RNN and has long-term memory functionality. Tan et al. [30] divided the voltage curve in the constant current charging phase into four parts, extracted nine HFs as input of LSTM for SOH prediction, and used gray relational analysis (GRA) to verify the effectiveness of HFs. These methods obtain the features of battery decay over time from battery aging sequence data, which can be called methods based on battery aging features. However, RNNs’ ability to extract battery cycle features is weak, so methods based on battery aging features also need to manually extract features to form sequence data.

## 3. Data

In this section, the definition of SOH, the source of data, and the pre-processing of data are explained in detail.

### 3.1. Definition of SOH

The SOH of the battery represents a stage in the battery life cycle. It evaluates the health of the current specific performance compared to the new state. The specific manifestations of SOH are diverse. In the existing research, researchers use characterization parameters such as capacity, impedance, or cycle number to evaluate health [24]. EMs have strong applicability, high accuracy, and strong interpretations. However, EMs usuall. The measurement of capacity is relatively simple and is the most widely used method at present. The formula is as follows:

(1) $$SOH=\frac{C_{aged}}{C_{fresh}}\times 100\%$$

where $C_{fresh}$ denotes the nominal capacity at a specific charging rate when the battery is in the initial state, $C_{aged}$ denotes the aging capacity measured at a specific time.

### 3.2. Source of Data

**Figure 1:**
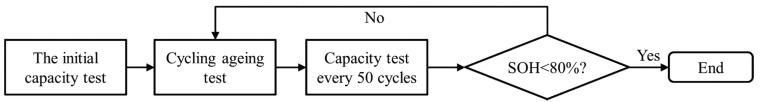
Flow chart of battery cycle test.

**Figure 2:**
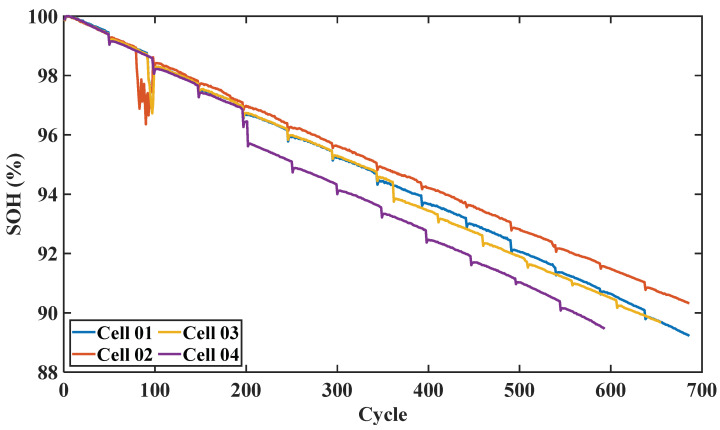
Aging track diagram of some batteries.

This paper uses 12 commercial 18,650 lithium batteries of the same model with a rated capacity of 4.8 Ah. In the aging test, the cycle data of the battery were collected through repeated charging, discharging, and impedance testing at 24 °C room temperature. The batteries were charged at a charge rate of $I_{c}$ in constant current mode until the voltage reached 4.2 V. Then, the constant voltage mode was used and the batteries were charged continuously until the charging current dropped to 15mA. After the charging process was completed, the battery returned to room temperature and began the discharge process. In constant current mode, the batteries are discharged at the discharge rate of $I_{d}$ until the voltage drops to 2.5 V. $I_{c}$ and $I_{d}$ of each battery are different, and other test conditions are identical. Before the aging cycle test of the battery, the initial capacity test shall be conducted to determine the actual rated capacity of each battery. Then, conduct a periodic capacity test at the cycle interval of every 50 full equivalent cycles (FEC), and the test flow is shown in Figure 1. The aging curve of some batteries is shown in Figure 2.

### 3.3. Data Pre-Processing

Generally, the discharge of the power battery shall meet the requirements of the vehicle, so the discharge process is unpredictable, and the charging process is relatively stable. Therefore, SOH prediction based on battery data during the charging process can be applied under most operating conditions. In all battery data, the voltage measurement is easier to measure. As a result, the voltage can be used as the reference to process other data. In the constant current charging stage, part of the charging voltage range $\left[V_{low},V_{high}\right]$ is defined, and the corresponding time period of this voltage interval $\left[t_{low},t_{high}\right]$ can also be obtained. The battery data of the corresponding period is interpolated into a vector of fixed length *L*. The matrix $M\in R^{L\;timesN}$ is formed by splicing vectors of different battery data, and *N* is the number of battery data types.

### 3.4. Structure of the Dataset

According to the above method, the data of 12 batteries with the same model and different experimental conditions were obtained. Two of them were left as the test set for this experiment. The remaining ten batteries were used to train the SOH prediction model.

In addition, the ten batteries used for training were randomly shuffled. Furthermore, 80% of the data were extracted as the training set, which was used to calculate the gradient and update the model parameters during training. The remaining 20% data was used as the verification set to calculate the generalization ability of the trained model during training. If necessary, the program was terminated in advance to prevent over fitting.

## 4. Methodologies

**Figure 3:**
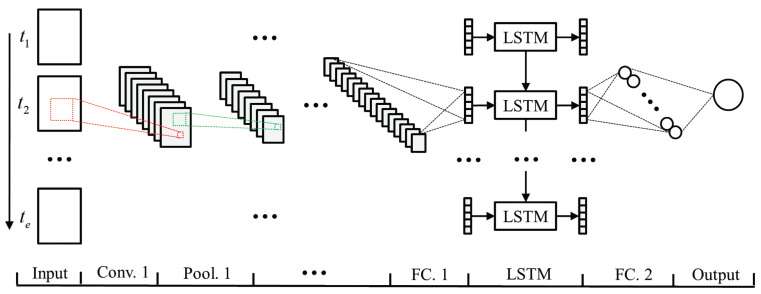
Overall structure of battery SOH prediction model based on multi-feature fusion.

In this paper, a SOH prediction model based on multi-feature fusion is proposed. The basic models of this model are CNN and LSTM, in which CNN obtains the battery cycle features from the input. The battery cycle features are input into LSTM to obtain the battery aging features according to the battery aging sequence. Finally, the predicted SOH value of the battery is obtained through the full connection layer. The overall architecture of the model is shown in Figure 3.

### 4.1. Convolutional Neural Network

**Figure 4:**
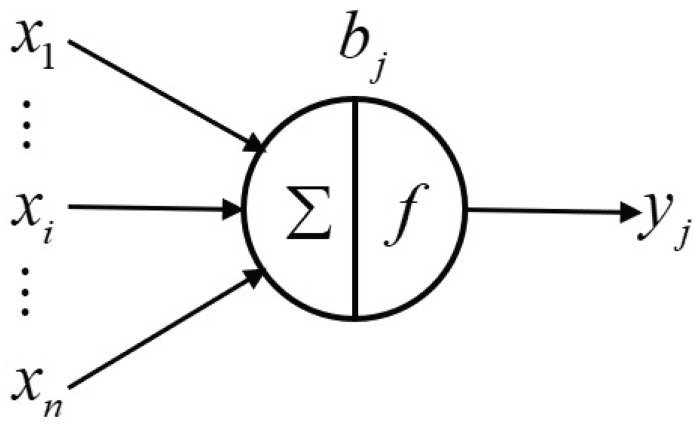
Structure of neurons.

Convolutional neural networks are multilayer artificial neural networks specially designed for processing two-dimensional input. Each layer of the network is composed of multiple two-dimensional planes. A convolution layer is composed of multiple feature maps. Each feature map is composed of multiple neurons. The neuron is the basic processing unit of the artificial neural network, which is generally a multi-input single-output unit. Its structural model is shown in Figure 4.

Where $x_{i}$ represents the input. *n* input signals were simultaneously input into the neuron *j*. $w_{ij}$ represents the weight value of the connection between the input signal and the neuron *j*. $b_{j}$ represents the bias of the neuron. $y_{j}$ is the output of the neuron *j*. The corresponding relationship between input and output can be expressed by the following formula:

(2) $$y_{j}=\sigma \left(b_{j}+\sum_{i=1}^{n}\left(x_{i}\times w_{ij}\right)\right)$$

where σ is the activation function, which can have many choices, including the rectified linear unit (ReLU), sigmoid function, tanh function, radial basis function, etc. [31].

**Figure 5:**
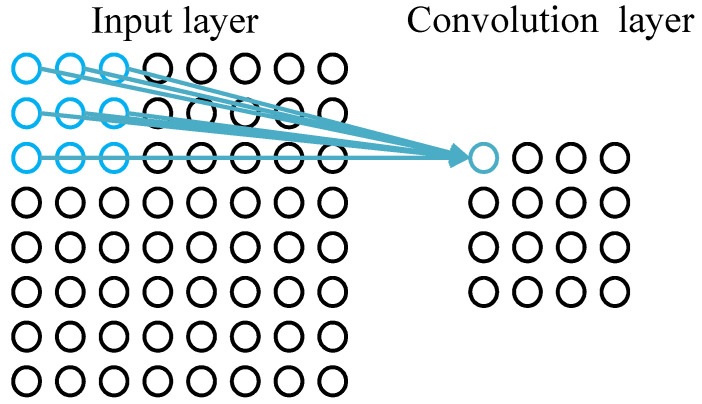
Operation process of convolution layer.

Each neuron of convolutional layer is connected with the local area of the upper layer’s feature map through the convolution kernel as shown in Figure 5. The convolution kernel is a weight matrix. The convolution layer of CNN extracts different features of input through convolution operation, the lower convolution layer extracts lower level local features, and the higher convolution layer extracts higher level global features. The convolution process is described as follows:

(3) $$a^{1}=\sigma \left(b+w*a^{0}\right)$$

where $a^{1}$ represents the output of a neuron in the hidden layer. $a^{0}$ represents the input of the hidden layer in the corresponding area of the neuron, *b* is the bias, σ is the activation function, ∗ represents the convolution operation, and *w* is the convolution weight matrix.

**Figure 6:**
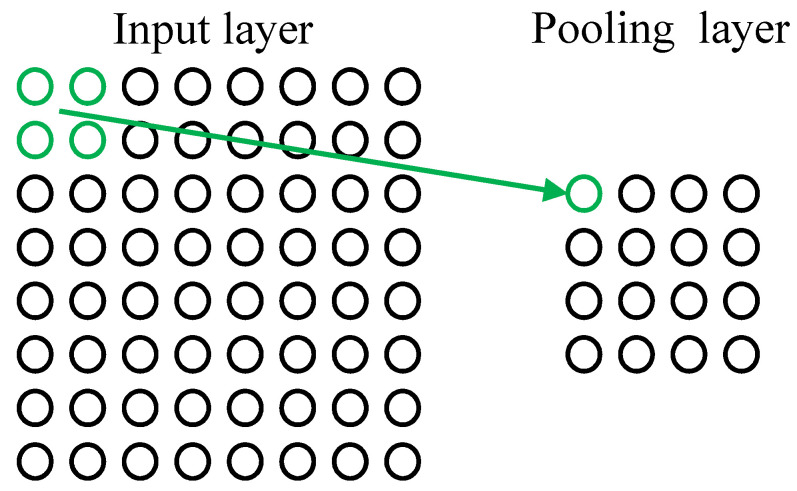
Operation process of pooling layer.

The neurons in the pooling layer are also connected to the local receptive fields of their input layer as shown in Figure 6. Generally, the local receptive fields of different neurons do not overlap. The pooling layer plays the role of secondary feature extraction, and each neuron of the pooling layer performs pooling operations on the local receptive region.

### 4.2. Long Short-Term Memory

**Figure 7:**
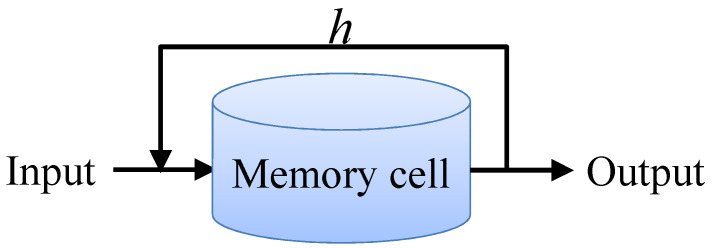
Structure of memory cell of RNN.

RNN is very effective for sequential data. It can mine temporal information and semantic information in data. This is because RNN can remember the information $h^{t}$ at each time. The output of neurons at each time is not only determined by the input $x^{t}$ at that time but also related to the hidden state $h^{t-1}$ at the previous time, as shown in Figure 7.

**Figure 8:**
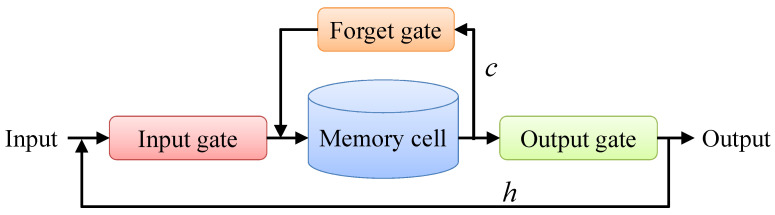
Structure of memory cell of LSTM.

However, the state of RNN’s memory cell is only simple short-term information, and the memory of long-term information is insufficient. LSTM adds a neuron state $c^{t}$ of the memory cell and three gating mechanisms based on RNN. This enables LSTM to selectively memorize historical information and grants it stronger long-term memory ability. The unit structure of LSTM is shown in Figure 8. The calculation process is as follows:

The input gate determines the information $\left(h^{t-1},x^{t}\right)$, which enters into memory cell at each time.

(4) $$g_{i}^{t}=\sigma \left(W_{i}\cdot \left[\begin{array}{c} x^{t}\\ h^{t-1} \end{array}\right]+b_{i}\right)$$

where $g_{i}^{t}$ is the input gate value of *t*, and $W_{i},b_{i}$ is the weight and bias of the input gate.

${\tilde{c}}_{t}$ is the pre-input state in which the result of the parameter processing of the input data $\left(h^{t-1},x^{t}\right)$ by $W_{c},b_{c}$. The result passes through tanh activation function and ${tildec}_{t}\in \left[-1,1\right]$.

(5) $${\tilde{c}}^{t}=\tanh\left(W_{c}\cdot \left[\begin{array}{c} x^{t}\\ h^{t-1} \end{array}\right]+b_{c}\right)$$

The forget gate determines the information be forgotten in the $c^{t-1}$ of the previous time in the memory cell at each time.

(6) $$g_{f}^{t}=\sigma \left(W_{f}\cdot \left[\begin{array}{c} x^{t}\\ h^{t-1} \end{array}\right]+b_{f}\right)$$

where $g_{f}^{t}$ is the forget gate value of *t* and $W_{f},b_{f}$ is the weight and bias of the forget gate.

The input gate controls the information of the pre-input state and the forget gate controls the information of the cell state $c^{t-1}$ at the last moment to obtain the cell state $c^{t}$ at each moment.

(7) $$c^{t}=g_{f}^{t}⊙c^{t-1}+g_{i}^{t}⊙{\tilde{c}}^{t}$$

where odot is the Hadamard product, representing the multiplication of corresponding elements in the matrix.

The output gate determines the information output from the memory cell at each time.

(8) $$g_{o}^{t}=\sigma \left(W_{o}\cdot \left[\begin{array}{c} x^{t}\\ h^{t-1} \end{array}\right]+b_{o}\right)$$

where $g_{o}^{t}$ is the output gate value of *t* and $W_{o},b_{o}$ is the weight and bias of the output gate.

Therefore, the output of the LSTM unit, that is, the hidden state $h^{t}$ at the current time, can be obtained by the following formula:

(9) $$h^{t}=g_{o}^{t}⊙\tanh\left(c^{t}\right)$$

### 4.3. Transfer learning

This study used a common method in the field of deep learning called transfer learning. Transfer learning helps model training of target tasks by transferring model parameters learned from source tasks. Transfer learning applies to two highly related tasks so that the model developed for the source task can be transferred to the new model developed for the target task.

**Figure 9:**
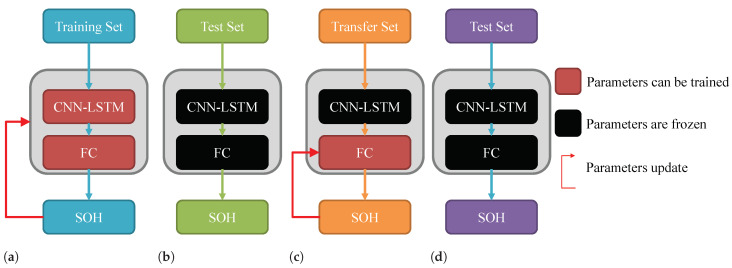
(**a**) Training process of source task. (**b**) Test process using pre-trained model directly. (**c**) Tuning process of transfer learning. (**d**) Testing process of transfer learning.

The source task of this paper is the SOH prediction for ten batteries, and the target task is the SOH prediction of the remaining two batteries. The data of the source task are used as the training set to train and update the parameters of the SOH prediction model, as shown in Figure 9a. Without using transfer learning, the target task data are used as the test set, and the SOH prediction results of the target task are obtained by freezing the trained model parameters, as shown in Figure 9b. When using transfer learning, a small amount of data at the beginning of the battery life cycle of the target task need to be taken as the transfer set. The CNN-LSTM layer is frozen and the transfer set is used to train and update the parameters of FC, which is also called fine-tuning, as shown in Figure 9c. The remaining data of the target task are used as a new test set. The test process is shown in Figure 9d.

## 5. Experiment

This experiment ran on a computer with an Intel Core processor i5-12600k CPU, NVIDIA GTX 1080Ti GPU, and 64 GB RAM. To evaluate the SOH prediction effect of various models, this paper uses root mean square percentage error (RMSPE), mean absolute percentage error (MAPE) and standard deviation of the error (SDE) as the evaluation criteria. The formula is as follows:

(10) $$RMSPE=\sqrt{\frac{1}{m}\sum_{i=1}^{m}{\left(\frac{y_{i}-{\hat{y}}_{i}}{y_{i}}\right)}^{2}}\times 100\%$$

(11) $$MAPE=\frac{1}{m}\sum_{i=1}^{m}\left|\frac{y_{i}-{\hat{y}}_{i}}{y_{i}}\right|\times 100\%$$

(12) $$SDE=\sqrt{\frac{1}{m}{\sum_{i=1}^{m}\left(x_{i}-\bar{x}\right)}^{2}}$$

where *m* is the number of data participating in the calculation of error. $y_{i}$ is the real value of SOH and ${\hat{y}}_{i}$ is the predicted value of SOH. $x_{i}=y_{i}-{\hat{y}}_{i}$ is the error of SOH, and $\bar{x}$ is the average value of the error.

### 5.1. Configuration of Experiment

The input matrix $M\in R^{100\times 14}$, which means that there are 14 types of battery data and each type of data is interpolated into 100 points. Grid search is used to obtain the optimal model parameters. Through loop traversal, the parameters with the highest accuracy in the verification set can be found within the specified parameter range. The parameters finally selected by CNN are shown in Table 1. The number of LSTM layers is five, the number of hidden neurons in the LSTM layer is 256, and the number of hidden neurons in FC.2 is 16.

### 5.2. Baseline

The baseline method compared in this paper is LSTM based on battery aging characteristics and three-layer CNN.

LSTM: In the constant current charging stage, the charging time when the voltage rises from 3.4 V to 4 V is the battery aging features [32]. The number of LSTM layers is five. The number of hidden neurons in LSTM layer is 256, and the number of hidden neurons in the full connection layer is 16.

CNN: In this paper, the battery data of multiple cycles are input into the CNN as the feature matrix of different channels. The size of convolution kernel in the first layer is (5,2), and that in the other two layers is (5,1). The stride is set to 1 without padding. The numbers of convolution kernels are 6, 16, and 32. Finally, it goes through two fully connected output layers.

### 5.3. Results of Experiment

The source task of this paper is the cyclic data of ten batteries, and the target task is the cyclic data of the remaining two batteries. The battery data of the source task are used to train the two models, respectively, and the trained parameters of the model are saved as the parameters of pre-trained model. Then, the pre-trained model is used to predict the target task as the test, and the results are shown in Table 2.

**Figure 10:**
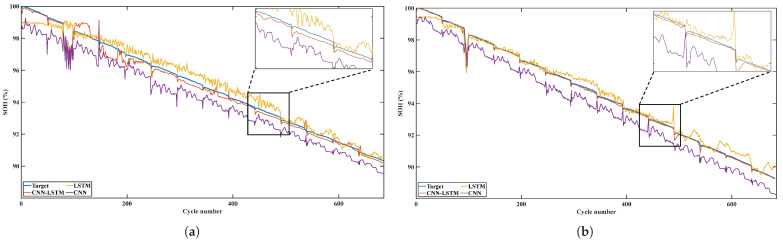

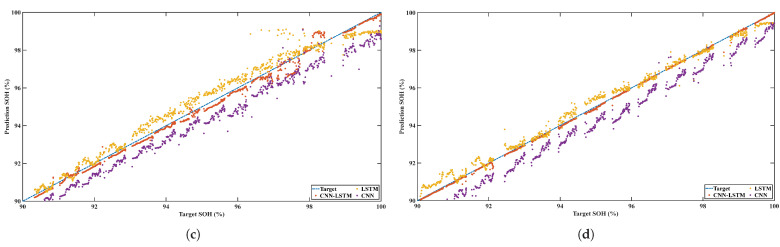
(**a**) Forecast results of Cell 2. (**b**) Forecast results of Cell 3. (**c**) Comparison between the predicted results and the true values of Cell 2. (**d**) Comparison between the predicted results and the true values of Cell 3.

Figure 10 shows the prediction results of two test batteries. It can be seen that although LSTM with battery charging time as its input feature can follow the trend of SOH decline, the prediction results are not stable enough and show obvious fluctuations. Especially at the beginning of the battery life cycle, the prediction results of the single input LSTM differ greatly from the target SOH. This is because, in the experiment, the charging and discharging rates of different batteries are different. When the working environment of the battery changes, the charging time of the fixed voltage segment is also affected by the charging rate. At the same time, the aging factors of the battery are diverse. Although the correlation between the charging time of the fixed voltage segment and SOH is strong, LSTM with the battery charging time as the input feature has a poor prediction effect. The fluctuation of the prediction results of the single CNN model is smaller than that of the single LSTM model. However, because the working conditions of the batteries in the training set and the batteries in the test set are different, there is a large deviation in all prediction results. When the fusion model is used for SOH prediction, the battery cycle features automatically extracted by CNN can closely follow the declining trend of SOH after passing through LSTM with the same structure.

**Figure 11:**
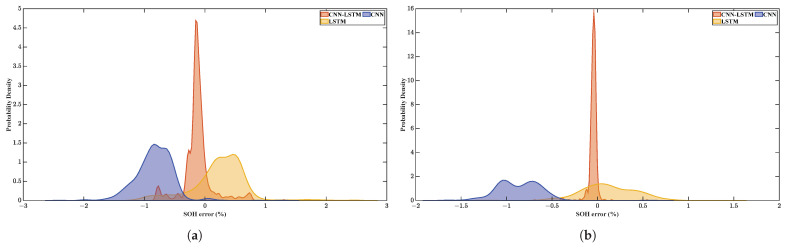
(**a**) The probability density diagram of errors of Cell 2. (**b**) The probability density diagram of errors of Cell 3.

The probability density diagrams of the errors of the different methods are shown in Figure 11. From the diagrams of the two batteries, it can be seen that the error distributions of LSTM model with charging time as the input feature and single CNN model are relatively dispersed, while the error distribution of CNN-LSTM fusion model is relatively concentrated. The position of the centralized trend of the three methods cannot be determined, and there is a certain deviation from the zero error position. Specifically, the position of the centralized trend of the CNN-LSTM fusion model is relatively closer to the zero point.

### 5.4. Results of Transfer Learning

From the experimental results, it can be seen that directly using the pre-trained model to test the target battery can also obtain results that meet the battery recession trend. However, there is an offset between the test results obtained in this manner and the actual target value. This is because the pre-trained model can obtain the cycle features and aging features of the target battery, but when mapping the battery features to the predicted SOH value, the full connection layer used is prone to overfitting. Therefore, the transfer learning method is used to tune the pre-trained model with the first 50 cycles of the test battery, and the prediction effect of three methods has been improved to a certain extent, as shown in Table 3. Among them, CNN has the greatest improvement effect, which also reflects the disadvantage of insufficient robustness of the method without transfer learning.

**Figure 12:**
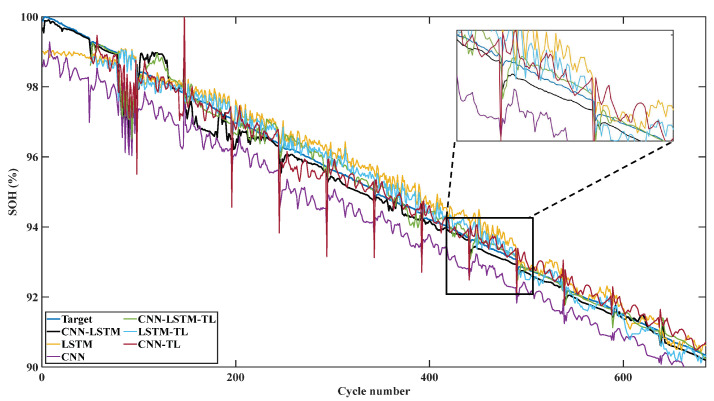
Forecast results and transfer results of Cell 2.

From the prediction results of Cell 2, as shown in Figure 12, it can be seen that the deviation of the three methods has been alleviated after transfer learning. However, the trend of the final prediction results is similar to that of using the pre-trained model directly. Therefore, LSTM of single charging time input and the single CNN model still have obvious fluctuation. Even the single CNN model has a great error every 50 cycles. In contrast, the CNN-LSTM fusion model maintained a good prediction trend and tuned the mapping relationship of the full connection layer, so the prediction results are closer to the target SOH value.

**Figure 13:**
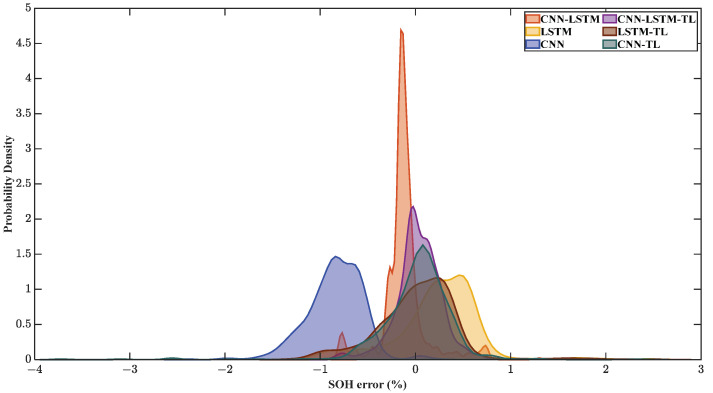
The probability density diagram of errors of Cell 2 after transfer learning.

The probability density diagram of the errors after transfer learning is shown in Figure 13. After transfer learning, the error of CNN-LSTM becomes more dispersed but still more concentrated than that of LSTM with single charging time input. The error distribution of LSTM with single charging time input is basically unchanged. However, the location of the concentration trend of the three methods is close to zero-error position, and the prediction results are better.

## 6. Conclusions

Battery feature acquisition is one of the main difficulties in SOH prediction. In this paper, a battery SOH prediction model based on multi-feature fusion is proposed. This model uses CNN to automatically extract the cycle features of the battery from the original battery data. The cycle features form the feature sequence of battery aging according to the sequence of battery aging and the aging features of the battery are obtained from the aging sequence by LSTM. Finally, a full connection layer is used to integrate battery features and output SOH prediction results. The results show that in the task of battery SOH prediction, the stability and accuracy of the proposed method are better than those of LSTM model with manually screened features and the single CNN model. In addition, this paper introduces transfer learning to further improve the effectiveness of the model prediction. The results show that when good battery features are obtained, tuning the full connection layer can effectively slow down the deviation of prediction results and improve the prediction accuracy of the model.

Although the deep learning method proposed in this paper shows good results, there are still some limitations to the online use of this method. First of all, the same type of lithium-ion battery is used in this paper, and there is less exploration of the applicability of the model. Secondly, the working environment of the selected battery is relatively simple, and the difference is only in the charge or discharge rate, which does not conform to the changeable working conditions in actual use. This will prompt us to further improve the data-driven SOH prediction method.

## Figures and Tables

**Table 1:** Configuration of CNN.

Layer	Conv.1	Pool.1	Conv.2	Pool.2	FC.1
Size of kernel	(5, 5)	(2, 2)	(5, 5)	(2, 1)	-
Number of kernels	6	-	16	-	-
Stride	(1, 1)	(2, 1)	(1, 1)	(2, 1)	-
Padding	0	0	0	0	-
Number of neurons	5760	2592	3520	1760	16

**Table 2:** Test results directly using the pre-trained model.

Battery	Cell 2			Cell 3		
Method	CNN-LSTM	LSTM	CNN	CNN-LSTM	LSTM	CNN
RMSPE	0.28%	0.53%	0.93%	0.24%	0.35%	1.62%
MAPE	0.21%	0.42%	0.88%	0.22%	0.28%	1.55%
SDE	0.010	0.019	0.013	0.004	0.014	0.020

**Table 3:** Test results of Cell 2 with transfer learning applied.

Battery	CNN-LSTM		LSTM		CNN	
Transfer learning	NO	YES	NO	YES	NO	YES
RMSPE	0.28%	0.24%	0.53%	0.45%	0.93%	0.43%
MAPE	0.21%	0.18%	0.42%	0.32%	0.88%	0.27%
SDE	0.011	0.010	0.020	0.019	0.013	0.018

## Data Availability

Not applicable.
